# Supplementary material for: A Rapid-Patterning 3D Vessel-on-Chip for Imaging and Quantitatively Analyzing Cell–Cell Junction Phenotypes
Source: Bioengineering (Basel). 2023 Sep 13;10(9):1080. doi: 10.3390/bioengineering10091080 (PMC10525190; doi:10.3390/bioengineering10091080)
Supplement: Supplementary file 1 [file bioengineering-10-01080-s001.zip › Supplemental materials.pdf]

Supplemental information for

A rapid-patterning 3D vessel-on-chip for imaging and quantitatively analyzing cell-cell junction phenotypes

Li Yan[1*#], Cole W. Dwiggins[1#], Udit Gupta, Kimberly M. Stroka[1,2,3,4*]

Table S1. Information of antibodies and fluorescent stains

Antibody	Vendor	Catalog number	Dilution
Anti-ZO-1	ThermoFisher	61-7300	1:200
Occludin	ThermoFisher	33-1500	1:200
Claudin-5	Abcam	Ab15106	1:200
Alexa Fluor™ 488 Phalloidin	ThermoFisher	A12379	1:40
Goat anti-Mouse IgG (H+L) Cross-Adsorbed Secondary Antibody, Alexa Fluor 568	ThermoFisher	A11011	1:1000
